# High Content Image Analysis as a Tool to Morphologically Distinguish Macrophage Activation and Determine Its Importance for Foamy Alveolar Macrophage Responses

**DOI:** 10.3389/fimmu.2021.611280

**Published:** 2021-09-01

**Authors:** Ewelina Hoffman, Paulina Napieralska, Rhamiya Mahendran, Darragh Murnane, Victoria Hutter

**Affiliations:** ^1^Centre for Topical Drug Delivery and Toxicology School of Life and Medical Sciences, University of Hertfordshire, Hatfield, United Kingdom; ^2^Department of Biochemistry and Molecular Diagnostics, Faculty of Pharmacy, Medical University of Lodz, Lodz, Poland

**Keywords:** alveolar macrophages, foamy alveolar macrophages, macrophage morphometrics, vacuolation, cytokine activation

## Abstract

**Introduction:**

Lung diseases are an increasing global health burden affecting millions of people worldwide. Only a few new inhaled medicines have reached the market in the last 30 years, in part due to foamy alveolar macrophage (FAM) responses observed in pre-clinical rat studies. The induction mechanism and signaling pathways involved in the development of highly vacuolated ‘foamy’ phenotype is not known. Furthermore, it has not been determined if these observations are adaptive or adverse responses.

**Aim:**

To determine if high content image analysis techniques can distinguish between alveolar macrophage activation (LPS/IFN-γ activated and IL-4 activated macrophages) and if this could be applied to understanding the generation of ‘foamy’ macrophage phenotypes.

**Methods:**

NR8383 rat alveolar macrophages were stimulated with a mix of cytokines (LPS/IFN-γ or IL-4) for 24 h. The cells were further exposed to FAM inducing-compounds amiodarone and staurosporine. Following 24 h incubation, phagocytosis and lipid accumulation were measured using flow cytometry and high content image analysis techniques. The alveolar macrophages responses after exposure to cytokines were assessed by evaluation: (i) cell surface and biochemical markers such as: nitric oxide production, arginase-1 activity and MRC-1 receptor expression (ii) cellular morphology (iii) cellular functionality (phagocytic activity and lipids accumulation).

**Results:**

Macrophages activated with LPS/IFN-γ showed distinct morphological (increased vacuolation) features and functionality (increased lipidosis, decreased phagocytic activity). Foamy macrophage phenotypes induced by amiodarone also displayed characteristics of proinflammatory macrophages (significantly increased nitric oxide production, increased vacuolation and lipidosis and decreased phagocytosis). In contrast, staurosporine treatment resulted in increased NO production, as well as arginase-1 activity.

**Conclusion:**

High content image analysis was able to determine distinct differences in morphology between non-activated and LPS/IFN-γ activated macrophages, characterized by increased vacuolation and lipidosis. When exposed to compounds that induce a FAM phenotype, healthy non-activated macrophages displayed proinflammatory (amiodarone) or pro-apoptotic (staurosporine) characteristics but these responses were independent of a change in activation status. This technique could be applied in early drug discovery safety assessment to identify immune responses earlier and increase the understanding of alveolar macrophage responses to new molecules challenge in development of new inhalation therapies, which in turn will enhance decision-making in an early safety assessment of novel drug candidates.

## Introduction

Alveolar macrophages are a heterogenous population of lung immune cells involved in health and disease (1). Their main function is provide tissue homeostasis by responding to pathogens, clearance of surfactant and cell debris and to modulate the adaptive immune response through antigen processing and presentation (1). Additionally, they are involved in the resolution of inflammation and tissue repair in the lungs (1). Alveolar macrophages dynamically alter their phenotype and function depending on their underlying microenvironment resulting in shifts in their polarization state between classically (M1) and alternatively (M2) activation states (2). M1-macrophages have the role of effector cells in cellular immune responses. They are typically activated by stimuli such as lipopolysaccharide (LPS) and interferon gamma (IFN-γ) which induce macrophages to produce large amounts of pro-inflammatory cytokines such as tumor necrosis factor alpha (TNF-α), interleukin (IL)-1-β, IL-6, IL-12 or IL-23 (3). The antimicrobial function of M1-macrophages is linked to up-regulation of enzymes, such as inducible nitric oxide synthase (iNOS) which in turn generates nitric oxide (NO) (4). In contrast, M2-macrophages promote tissue repair and remodeling and have been reported to be involved in tumor progression. M2-macrophage polarization is induced mainly (but not limited only to) by interleukin 4 (IL-4) and results in the higher activity of arginase-1 enzyme, higher expression of MRC-1 receptor, as well as in the production of high levels of IL-10 or IL-8 (3). Alveolar macrophages alter their polarization state, from immune effector cells to the wound-healing cells, in response to the surrounding microenvironment. The plasticity of alveolar macrophages to alter their polarization state is reported to be altered in certain pathological conditions, [e.g. chronic obstructive pulmonary disease (COPD)], which restricts their functional capabilities (5).

A foamy alveolar macrophage (FAM) is a term used to describe a highly vacuolated phenotype of alveolar macrophages viewed under light microscope (6, 7). This ‘foamy’ phenotype is commonly observed during non-clinical studies of new medicines for inhalation and is likely to represent a spectrum of adverse and adaptive responses (6). It is not well understood whether FAM responses are adverse or adaptive, and there are no acceptance criteria to differentiate between various types of FAM phenotypes. Furthermore, the induction mechanism and signaling pathways involved in the development of a highly vacuolated phenotype has not been explored in detail. FAM can be induced *via* different pharmacological or non-pharmacological mechanisms (8). Currently, there are two established distinct mechanisms which observe development of a FAM phenotype, namely phospholipidosis or apoptosis, which can be induced either by amiodarone or staurosporine respectively (9, 10). Whilst the cellular and morphometric status of these FAM have been reported previously (9), no studies have investigated the role that macrophage activation status may play in FAM generation and response. This role may be of dual nature; pre-existing M1 or M2 populations of macrophages may contribute to FAM responses and/or FAM inducers may induce M1 or M2 polarization.

The overall aim of this study was to evaluate if high content image analysis methodologies which assess morphology and cell health within early safety assessment could be used to ascertain macrophage polarization/activation and provide an earlier indication of potential immune responses and/or foamy alveolar macrophage induction in the drug discovery process. In order to achieve this, *in vitro* rat alveolar macrophages were profiled (including polarization status, phagocytosis capability and lipid accumulation) in response to established foamy macrophage inducers, amiodarone and staurosporine. We hypothesize that specific FAM phenotypes may be associated with differential polarization status which may affect the resolution of the FAM phenotype and facilitate early decision-making in drug discovery process.

## Materials and Methods

### Materials

Amiodarone hydrochloride 5 µM (cat#A8423, Sigma Aldrich, Pool, Dorset, UK) and staurosporine 0.1 µM (cat#S4400, Sigma Aldrich, Pool, Dorset, UK) were used to induce foamy macrophage phenotypes without significantly affecting the cell count and viability. Lipopolysaccharide (LPS, cat#L2630, Sigma Aldrich, Dorset, UK), recombinant rat interferon gamma (IFN-γ, cat#550072), and recombinant rat interleukin 4 (IL-4, cat#555107) were purchased from BD Biosciences, (Wokingham, Berkshire, UK), while recombinant rat interleukin 13 (IL-13, cat#CB243IL13) was purchased from PAN-Biotech (Wimborne, Dorset, UK). LPS and interleukins were used to activate macrophages to M1 or M2 polarization state.

### Cell Culture

The NR8383 cell line was chosen for this study as it provides a homogenous source of highly responsive alveolar macrophages (11, 12). Moreover, it is an established and well characterized alveolar macrophage cell line with appropriate culture characteristics and suitable as comparator cell line for *in vivo* alveolar macrophage responses observed in the Sprague-Dawley rat model (11, 12). The cell line was obtained from LGC Standards (cat#AgC11x3A, Teddington, Middlesex, UK) and used between passage numbers 2 and 20 from purchase. NR8383 cells were maintained in a humidified atmosphere at 37°C with 5% v/v CO_2_. Culture medium consisted of Kaighn’s modified Ham’s F12 medium (K-F12; cat# 21127030, Gibco, Life Technologies, UK) supplemented with 15% v/v heat-inactivated fetal bovine serum (FBS; cat#F7524, Sigma Aldrich, Dorset, UK), with 100 IU/ml penicillin-100 µg/ml streptomycin solution (cat#P4333, Sigma Aldrich, Dorset, UK) and 2 mM L-glutamine (cat#G7513, Sigma Aldrich, Dorset, UK). Cells were routinely sub-cultured when 70-80% confluent and seeded in multi-well plates.

### NR8383 Activation and Compound Exposure

NR8383 cells were seeded onto 96-well plates (cat#7340023, VWR International Ltd., Lutterworth, Leicestershire, UK) or 24-well plates (cat#7340020, VWR International Ltd., Lutterworth, Leicestershire, UK) at an optimal density of 0.5 x 10^5^ cells/well or 2.4 x 10^5^ cells/well respectively and cultured for 24 h. Three dosing schemes representing three *in vitro* models were explored. Model M0 represented healthy, non-activated macrophages to evaluate cell activation and function following 24 h exposure to amiodarone (5 µM) and staurosporine (0.1 µM). Model M(LPS+/IFN-γ+) represented proinflammatory activated macrophages (often found in asthma or COPD). M(LPS+/IFN-γ+) macrophage responses to the FAM inducers were evaluated. To this purpose, NR8383 were exposed for 24 h to100 ng/mL LPS and 20 ng/mL IFN -γ prior to exposure to amiodarone (5 µM) and staurosporine (0.1 µM) for an additional 24 h. Model M(IL-4+) represented IL-4-stimulated macrophages (often found in pulmonary fibrosis or lung tumor). M(IL-4+)-activated macrophage responses to the FAM inducers were evaluated. To this purpose, NR8383 were exposed for 24 h to 25 ng/mL IL-4 with or without 10 ng/mL IL-13 prior to exposure to amiodarone (5 µM) and staurosporine (0.1 µM) for an additional 24 h. At the end of the treatment period cells were evaluated for nitric oxide production, arginase-1 activity and MRC-1 receptor expression, phagocytosis and phospholipids accumulation.

### Cell Vacuolation and Quantification of Lipids

Fluorescence staining and imaging were performed as described previously by Hoffman et al. to determine the cellular lipid content and to assess macrophage morphology (9). Briefly, macrophages were incubated simultaneously with the tested compound and HCS LipidTox Phospholipid Red dye (cat#H34351, Invitrogen, Renfrewshire, UK) diluted 1:1000 (according to the manufacturer’s protocol) for 24 h. After the desired compound exposure time, the cells were fixed with 3.7% w/v paraformaldehyde (PFA; cat#P6148, Sigma Aldrich, Pool, Dorset, UK) containing Hoechst 33342 (10 µg/mL; cat#H3570, Invitrogen, Renfrewshire, UK) for 20 min, followed by one wash step with 100 µl phosphate buffered saline (PBS; cat#P4417, Sigma Aldrich, Pool, Dorset, UK). The cells were then incubated with HCS LipidTox Neutral Lipid Green dye (cat#H34475, Invitrogen, Renfrewshire, UK) diluted 1:1000 (according to the manufacturer’s protocol) and Cell Mask Deep Red (cat#H32721, Invitrogen, Renfrewshire, UK) diluted 1:1000 (according to the manufacturer’s protocol) for 30 min at room temperature for assessment of neutral lipid accumulation and morphometric characterization, respectively. The cells from the assay were stored in the dark at 4°C before sample acquisition.

Cell health (mitochondrial activity and cell membrane integrity) was assessed as outlined previously by Hoffman et al., 2017 (9). In brief, cells were either incubated with amiodarone or staurosporine. Following 24 h incubation, cells were stained with a dye cocktail containing Hoechst 33342 (10 µg/mL), MitoTracker Red (300 nM; cat#M7512, Invitrogen, Renfrewshire, UK) and Image-It-Dead Green (25 nM; cat#I10291, Invitrogen, Renfrewshire, UK) for 30 min. Cells were washed once with PBS and fixed with 3.7% PFA for 15 min. Cells were washed once before imaging.

Images were captured using the InCell Analyser 6500HS (GE Healthcare, Little Chalfont, Bucks, UK) with a 40x objective in standard 2D imaging mode with an exposure time of 0.1 s. A high content image analysis protocol developed previously by Hoffman et al. was employed (9). Briefly, InCell Developer Toolbox v 1.9.2, Level 3 software (GE Healthcare, Little Chalfont, Bucks, UK) was used to quantify parameters including: cell count, average cellular and nuclear area, average number of vacuoles per cell, average area of a single vacuole, average area of all vacuoles, percent of cell occupied by all vacuoles and intracellular lipid content. Nucleated cells were identified based on Hoechst 33342 staining. Cell Mask Deep Red dye was used to highlight the cytoplasmic regions with the cells, while negative staining with the dye was used to segment vacuoles within cells. The intercellular phospholipid and neutral lipid accumulation were quantified and reported as fluorescence intensity values of HCS LipidTox Phospholipid Red and HCS LipidTox Neutral Lipid Green stains.

### Nitric Oxide (NO) Quantification

The supernatant was collected, and nitric acid production was determined in the form of total nitrites using the Greiss Reagent System (cat#G2930, Promega, Southampton, Hampshire, UK) according to the manufacturer’s protocol. Briefly, 50 µl of supernatant was mixed with 50 µl of sulfanilamide solution (1% w/v sulfanilamide in 5% w/w phosphoric acid). Following a 10 min incubation, 50 µl of NED solution (0.1% w/v N-1-napthylethylenediamine dihydrochloride in water) was added and incubated for a further 10 min. The absorbance was measured at 540 nm, and the concentration of nitrite was calculated from a standard curve of sodium nitrite.

### Arginase Quantification

Arginase activity of the NR8383 macrophage lysates was measured by a colorimetric Arginase Activity Assay Kit (cat#MAK112, Sigma Aldrich, Pool, Dorset, UK) according to the manufacturer protocol. Briefly, substrate buffer (containing MnCl_2_ and L-arginine) was added to the lysates and incubated for 2 h at 37°C. The reaction was stopped by adding the urea solution for 1 h at room temperature. The urea generated by arginase was quantified by absorbance measurement at 430 nm. One unit of arginase activity is defined as the amount of Mn^2+^ activated enzyme that produces 1 μmol of ornithine/min at 37°C.

### MRC-1 Receptor Expression

MRC-1 receptor expression was quantified using flow cytometry. At the end of treatment period, cells were harvested, blocked with 10% goat serum (cat#ab7481, Abcam, Cambridgeshire, UK) and incubated with MRC-1 polyclonal antibody (cat#PA5-114370, Invitrogen, Renfrewshire, UK) for 30 min at room temperature. The cells were then labelled with Goat anti-Rabbit IgG (H+L) cross-absorbed secondary antibody, FITC (cat#F-2765, Invitrogen, Renfrewshire, UK) for 30 min at room temperature, in the dark. A representative 10,000 cells were acquired and analyzed using the Guava EasyCyte system (Guava EasyCyte 8HT, Millipore, UK). Cells were identified from free particles and cellular debris by their forward and side scatter. MRC-1 surface receptor expression was quantified by processing the green fluorescence (Ex/Em: 505/515) of cells and comparison with non-stimulated control.

### Phagocytosis

To determine phagocytic activity, NR8383 cells were incubated with 1.0 µm microspheres at a ratio of 1:30 (cells: particles). Briefly, at the end of treatment period, the media containing 1.0 µm Carboxylate-Modified Microspheres (cat#F8823, Invitrogen, ThermoFisher, UK) was added for 2 h and cells were incubated at normal culture conditions. Cells were harvested by gentle scraping and the cell fluorescence (Ex/Em: 505/515) was measured using the Guava EasyCyte system (Guava EasyCyte 8HT, Millipore, UK). Cells were identified from free particles and cellular debris by their forward and side scatter. Phagocytic activity was assessed by processing the green fluorescence (525 ± 30 nm) of cells and comparison with untreated control. At least 5000 cells were counted for each sample.

### Statistical Analysis

All experiments were repeated independently at least three times, with n=3 wells per experiment, and approximately 1000 – 2000 individual cells measured per well in the morphology study. Data from repeated experiments were assessed using the Shapiro-Wilk test for normality and found to fit a normal (Gaussian) distribution). All reported parameters were normalized to the number of viable cells in a sample. Statistical comparisons were made using a one-way ANOVA analysis with Bonferroni’s multiple comparison post-hoc test. Values of *p* < 0.05 were considered to be statistically significant and denoted as follows: *, *p* < 0.05; **, *p* < 0.01; and ***, *p* < 0.001.

## Results

### Confirmation of Macrophage Activation

**Figure 1:**
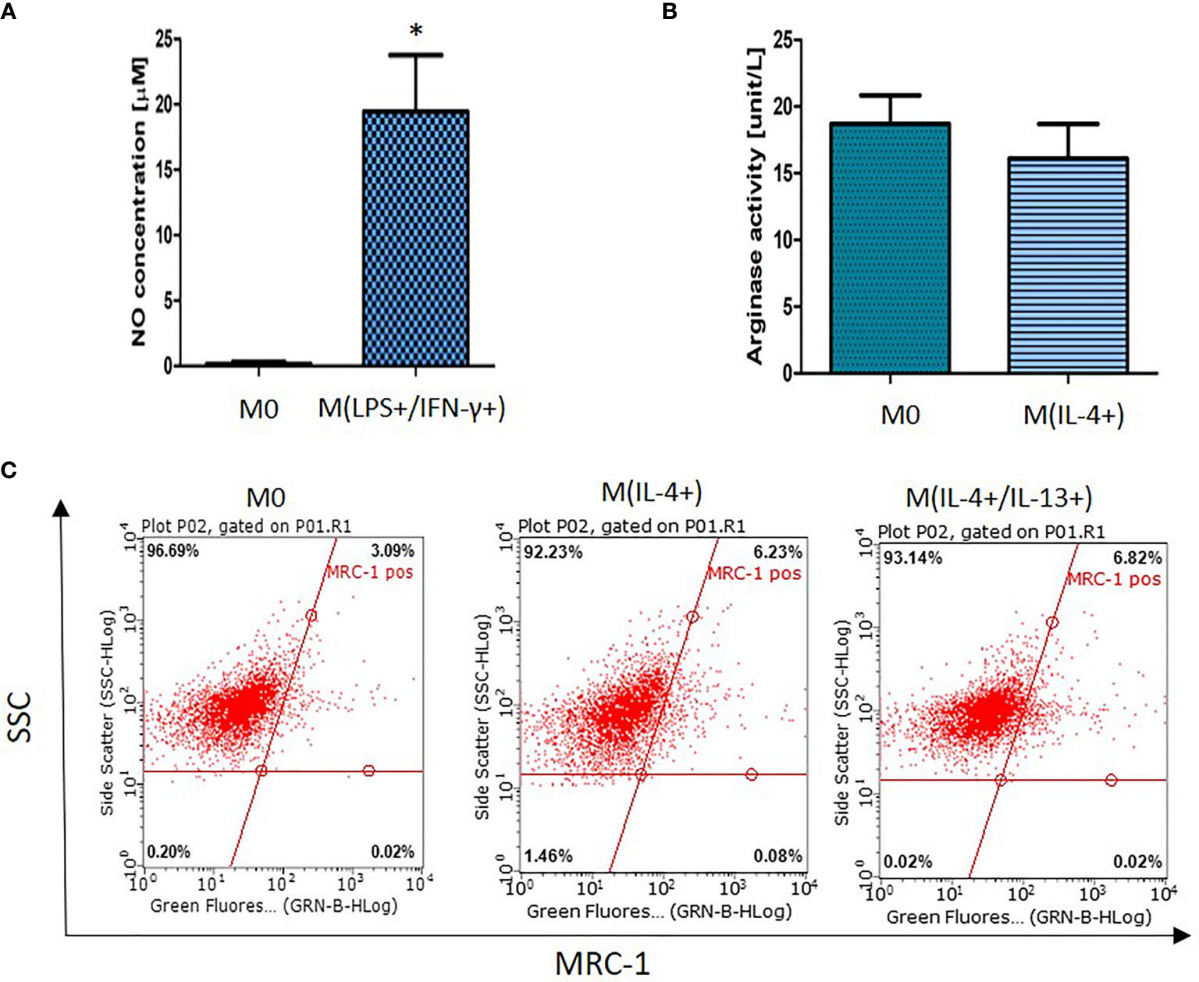
NR8383 rat macrophage activation status after stimulation with **(A)** LPS and IFN-γ, **(B)** IL-4 or **(C)** IL-4 or IL-4/IL-13. Data are normalized to cell count and presented as mean values ± SEM of three separate experiments. Statistical comparisons are made using a one-way ANOVA analysis with Bonferroni’s multiple comparison post-hoc test. Results are compared to non-polarized control (M0) and statistical significance is marked as follows: * indicates *p <* 0.01. **(C)** Representative fluorescent dot (quadrants) plots showing percentage of cells expressing MRC-1 surface receptor.

Macrophage activation was determined using a combination of cell surface and biochemical markers (4, 13). LPS/IFN-γ-activated macrophages [M(LPS+/IFN-γ+)] produced a significantly higher concentration of nitric oxide (NO) (*p* < 0.01) in comparison with non-activated macrophages which produced very low levels of NO (**Figure 1A**). Increased NO-production by LPS/IFN-γ-stimulated macrophages suggests classically (M1) activation. However, these findings should be supported by assessment of additional M1 polarization markers. The upregulation in arginase-1 (Arg-1) activity and MRC-1 receptor expression are considered as established markers for alternative (M2) activation (14). In this study NR8383 activation with IL-4 did not generate increased arginase activity (**Figure 1B**) suggesting that IL-4 was unlikely to have activated NR8383 cells. This finding was further supported by a non-significant increase in MRC-1 receptor expression (**Figure 1C**). Moreover, stimulation with a combination of IL-4 and IL-13 did not result in a distinct activation status.

### Distinct Morphological Features Identified for M(LPS+/IFN-γ+) Macrophages

**Figure 2:**
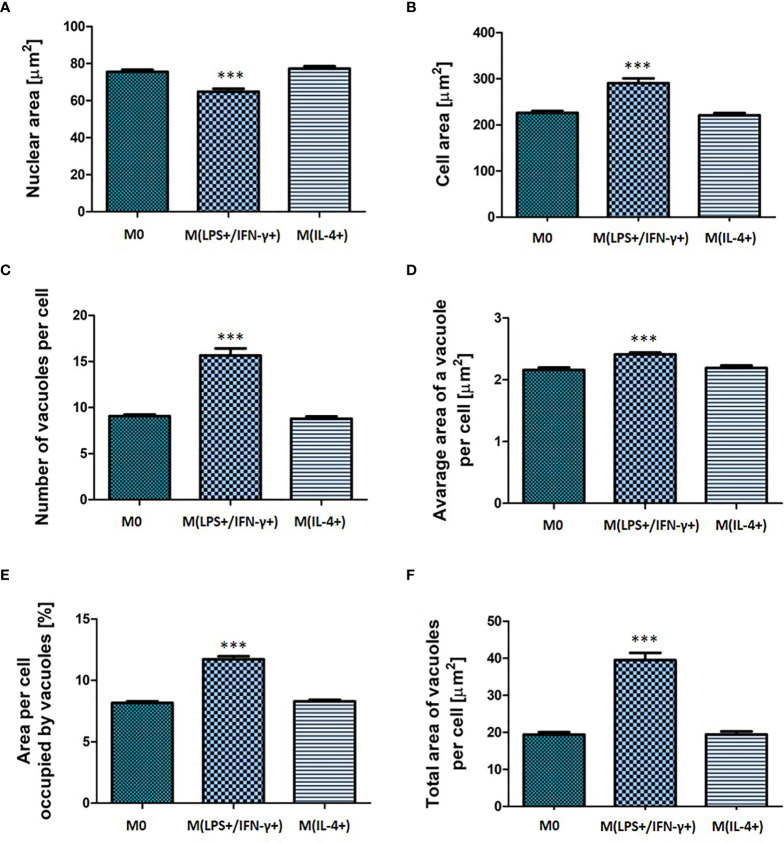
Morphological characteristic of non-activated, M(LPS+/IFN-γ+) or M(IL-4+) activated NR8383 rat macrophages. Nuclear area **(A)**, cellular area **(B)**, number vacuoles per cell **(C)**, average area of a single vacuole **(D)**, cellular area occupied by all vacuoles **(E)** and total area of all vacuoles per cell **(F)** were quantified in non-activated macrophages (M0), activated with 100 ng/mL LPS and 20 ng/mL IFN-γ [M(LPS+/IFN-γ+)] or activated with 30 ng/mL IL-4 [M(IL-4+)]. Data are presented as mean values ± SEM of three separate experiments. Statistical comparisons are made using a one-way ANOVA analysis with Bonferroni’s multiple comparison post-hoc test. Results are compared to non-polarized control (M0); *** indicates *p <* 0.001.

Macrophages were activated using a mix of LPS and IFN-γ, or IL-4, respectively. The cell morphology (nuclear and cellular area, number and size of vacuoles, area of a cell occupied by vacuoles) was assessed 24 h after activation (**Figure 2**). It was observed that LPS/IFN-γ activated cells displayed distinct morphological changes (*p* < 0.001) when compared to untreated cells. M(LPS+/IFN-γ+) macrophages displayed significantly increased (*p* < 0.01) total cell area from 226 (± 20) µm^2^ (M0) to 290 (± 51) µm^2^ [M(LPS+/IFN-γ+)] and significantly decreased (*p* < 0.01) nuclear area from 75 (± 5) µm^2^ (M0) to 65 (± 8) µm^2^ [M(LPS+/IFN-γ+)] when compared to non-polarized cells (**Figures 2A, B**). Moreover, M(LPS+/IFN-γ+) cells contained on average 6 (± 2) more vacuoles (*p* < 0.001), and the average area these vacuoles occupied increased from 2.16 (± 0.2) µm^2^ (M0) to 2.4 (± 0.2) µm^2^ [M(LPS+/IFN-γ+)] (*p* < 0.001) (**Figures 2C, D**). The total area of the cell occupied by vacuoles was 40 (± 9.5) µm^2^ in M(LPS+/IFN-γ+)-activated macrophages compared with only 20 (± 3.3) µm^2^ in non-polarized cells (*p* < 0.001) (**Figure 2F**). The M(LPS+/IFN-γ+) cell area occupied by vacuoles also significantly increased (*p* < 0.001) from 8.2 (± 0.7) % (M0) to 11.7 (± 1.2) % (M(LPS+/IFN-γ+)) (**Figure 2E**). None of the morphological features assessed for IL-4 treated cells were significantly different (*p* > 0.05) from non-polarized macrophages confirming that NR8383 activation to an M2 phenotype with IL-4 alone was likely to be unsuccessful. Percentage of alveolar macrophage population displaying increased/decreased features were calculated and presented as a heatmap in supplementary material (**Supplementary Figure 2**).

**Figure 3:**
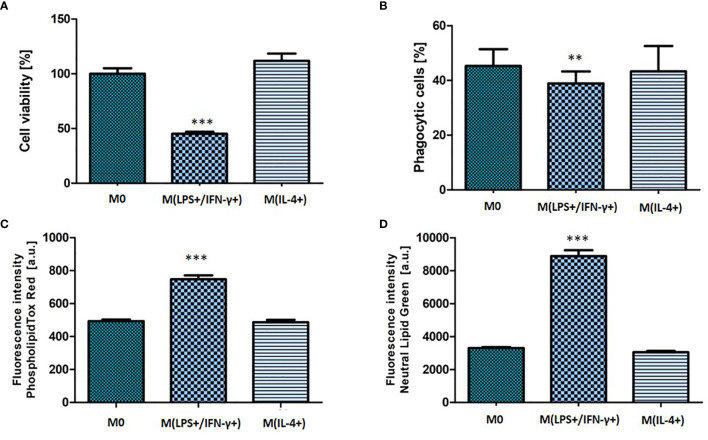
Functional characteristic of non-activated, M(LPS+/IFN-γ+) or M(IL-4+) activated NR8383 rat macrophages. Cell count **(A)**, phagocytic activity **(B)**, cellular phospholipid **(C)** and cellular neutral lipid content **(D)** were quantified in non-activated macrophages (M0), activated with 100 ng/ml LPS and 20 ng/ml IFN-γ [M(LPS+/IFN-γ+)] or activated with 30 ng/ml IL-4 [M(IL-4+)]. Data are normalized to cell count and presented as mean values ± SEM of three separate experiments. Statistical comparisons are made using a one-way ANOVA analysis with Bonferroni’s multiple comparison post-hoc test. Results are compared to non-polarized control (M0); ** indicate *p* < 0.01, *** indicate *p <* 0.001.

M(LPS+/IFN-γ+) activation phenotype was also reflected in NR8383 functionality (**Figure 3**). The percentage of cells that remained adhered to the well plate was calculated from the number of nucleated, polarized cells as a fraction of the adhered cells in the non-polarized (M0) control population. The fraction of adherent cells decreased to 45 (± 9) % of the non-polarized control (*p* < 0.001) (**Figure 3A**). There was a significant decrease (*p <* 0.01) observed in the phagocytic activity of macrophages with the phagocytic cell population decreasing from 51 (± 5.9) % (M0) to 41 (± 7.6) % (M(LPS+/IFN-γ+)) (**Figure 3B**). M(LPS+/IFN-γ+) cells had significantly accumulated phospholipids (1.5-fold increase; *p* < 0.001) and neutral lipids (2.7-fold increase; *p* < 0.001) in comparison to non-polarized M0 cells. Consistent with the finding of unchanged morphological features in NR8383 macrophages, no changes in macrophage function were observed following stimulation with IL-4.

### Amiodarone and Staurosporine Increase NO Production by Macrophages

**Figure 4:**
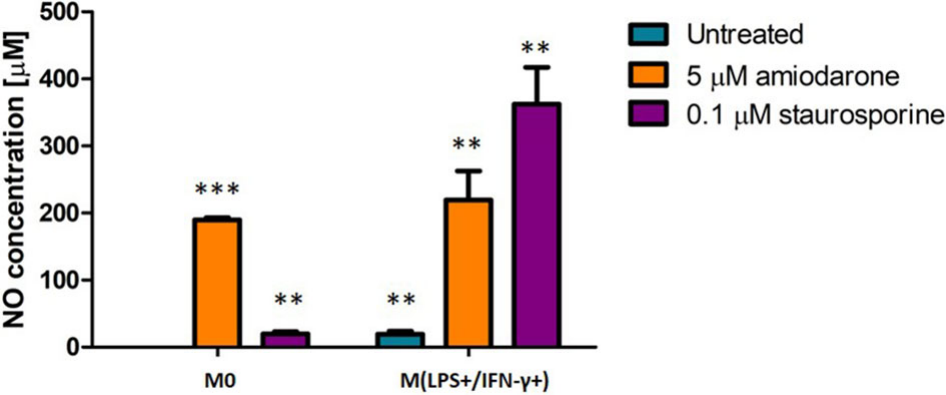
Effect on NO production in non-activated (M0) and LPS/IFN-γ activated NR8383 rat macrophages after 24 h exposure to 5 µM amiodarone (orange bars) and 0.1 µM staurosporine (purple bars). Data are normalized to cell count and presented as mean values ± SEM of four separate experiments. Statistical comparisons are made using a one-way ANOVA analysis with Bonferroni’s multiple comparison post-hoc test. Results are compared to non-polarized control (M0); **indicate *p* < 0.01 and *** indicates *p <* 0.001.

The ability of macrophages to undergo LPS/IFN-γ-activation and respond to a drug challenge was assessed. Nitric oxide (NO) production was quantified. Significant differences (*p* < 0.01 and *p* < 0.001) in NO production were observed dependent on whether the cells were activated with LPS/IFN-γ or not prior to a drug challenge (**Figure 4**).

The production of NO in untreated but activated macrophages M(LPS+/IFN-γ+) was significantly (*p* < 0.01) higher when compared to non-activated control cells (M0). After challenging both macrophage groups (M0 and M(LPS+/IFN-γ+)) with amiodarone, there was no significant (*p* > 0.05) difference in NO production between activated and non-activated macrophages. Whilst staurosporine treatment resulted in a significant (*p* < 0.01) increase in NO production by macrophages which were M(LPS+/IFN-γ+)-activated prior to a drug challenge. These findings suggest that M(LPS+/IFN-γ+)-polarized cells are more sensitive to exposure of apoptotic agents such as staurosporine.

### Staurosporine Increases Arginase Activity but Not MRC-1 Surface Receptor Expression

**Figure 5:**
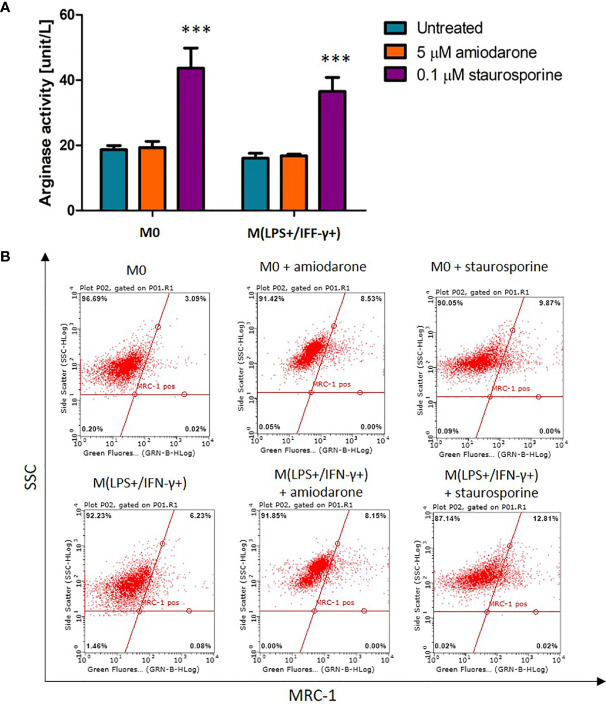
Effect on arginase activity and MRC-1 receptor expression in M0-non-activated and IL-4 activated NR8383 rat macrophages after 24 h exposure to 5 µM amiodarone and 0.1 µM staurosporine. **(A)** Effect on arginase activity. Data are normalized to cell count and presented as mean values ± SEM of three separate experiments. Statistical comparisons are made using a one-way ANOVA analysis with Bonferroni’s multiple comparison post-hoc test. Results are compared to non-polarized control (M0); *** indicates *p <* 0.001. **(B)** Representative fluorescent dot (quadrants) plots showing percentage of cells expressing MRC-1 surface receptor.

Arginase activity and MRC-1 surface receptor expression were measured as markers for alternatively activated macrophages (**Figure 5**). Although the NR8383 polarization to an M2 phenotype was not reflected in arginase-1 activity nor MRC-1 expression, a significant (*p* < 0.001) increase of arginase-1 activity was observed after exposure to 0.1 µM staurosporine. M0 and IL-4 treated macrophages stimulated with staurosporine showed a significant increase in arginase activity when compared to non-polarized cells (*p* < 0.001) (**Figure 5A**), whilst there was no significant increase (*p* > 0.05) in surface expression of the MRC-1 receptor (**Figure 5B**).

### Morphological Changes of M(LPS+/IFN-γ+)-Macrophages Are Not Modulated by FAMs Inducers

**Figure 6:**
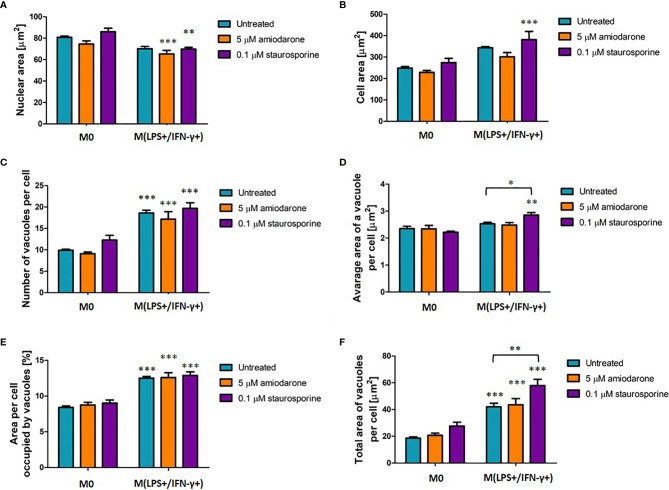
Morphological characteristic of M0 and LPS+/IFN-γ+ activated NR8383 rat macrophages after 24 h exposure to 5 µM amiodarone (orange bars) and 0.1 µM staurosporine (purple bars). Nuclear area **(A)**, cellular area **(B)**, number vacuoles per cell **(C)**, average area of a single vacuole **(D)**, cellular area occupied by all vacuoles **(E)** and total area of all vacuoles per cell **(F)** were quantified in non-polarized macrophages (M0) and LPS+/IFN-γ activated [M(LPS+/IFN-γ+)] cells followed by 24 h activation with FAM. Data are normalized to number of cells and presented as mean values ± SEM of three separate experiments. Statistical comparisons are made using a one-way ANOVA analysis with Bonferroni’s multiple comparison post-hoc test. Results are compared to non-polarized control (M0) (except otherwise indicated) and statistical significance is marked as follows: * indicate *p <* 0.05, ** indicate *p <* 0.01, *** indicate *p <* 0.001.

M0 and M(LPS+/IFN-γ+) macrophages were exposed to FAM inducers: 5 µM amiodarone and 0.1 µM staurosporine. Following 24 h incubation macrophage morphometrics was assessed (**Figure 6**). There were no significant changes (*p* > 0.05) observed in the non-polarized macrophage population (M0) regardless of the drug treatment. Noteworthy changes were observed in untreated M(LPS+/IFN-γ+)-polarized cells confirming their M(LPS+/IFN-γ+)-distinct appearance presented above in **Figure 2**. Additional treatment with FAM inducers did not change their morphological characteristics, with an exception of staurosporine stimulation, which significantly (*p* < 0.05 and *p* < 0.01) affected only two features: average area of a vacuole (**Figure 6D**) and total area of vacuoles (**Figure 6F**). Additionally, percentage of macrophage population displaying increased/decreased features were calculated in presented as macrophage response profiles in supplementary material (**Supplementary Figure 3**).

### Phagocytic Capability of NR8383 Is Not Affected by FAM-Inducers

**Figure 7:**
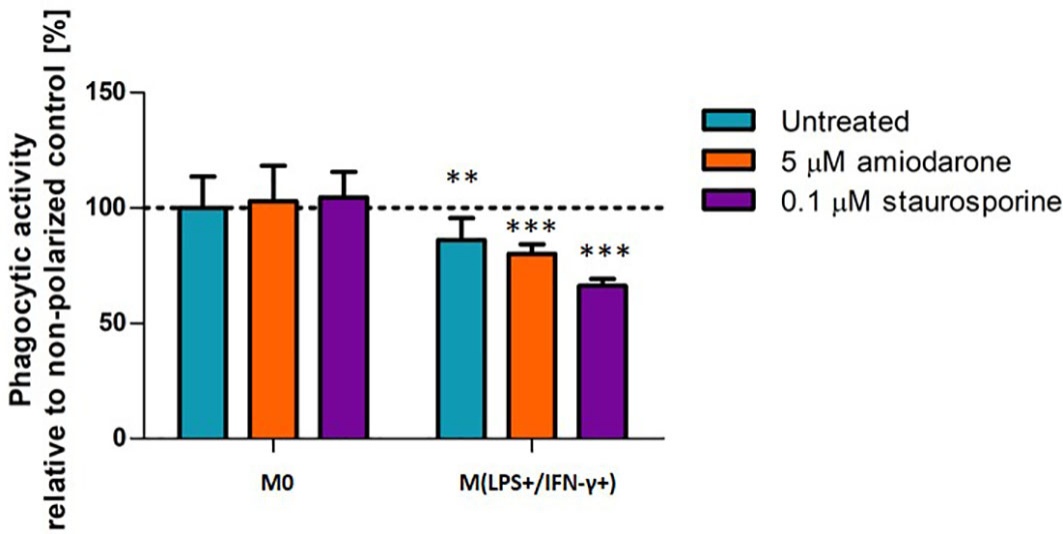
Phagocytic activity in M0-non-activated, LPS/IFN-γ or IL-4 activated NR8383 rat macrophages after 24 h exposure to 5 µM amiodarone (orange bars) and 0.1 µM staurosporine (purple bars). Data are expressed as a percentage of the phagocytic activity of non-polarized untreated NR8383 cells. Data represent the mean values ± SEM of three separate experiments. Statistical comparisons are made using a one-way ANOVA analysis with Bonferroni’s multiple comparison post-hoc test. Results are compared to non-polarized control (M0); ** indicate *p* < 0.01, *** indicates *p <* 0.001.

The phagocytic activity of M(LPS+/IFN-γ+) activated and/or foamy-induced macrophages was evaluated by incubation of the NR8383 cells with 1 µm microspheres for 2 h (**Figure 7**). The results were compared with non-polarized macrophages (M0). Neither amiodarone nor staurosporine affected phagocytic capability of non-polarized (M0) macrophages. M(LPS+/IFN-γ+) cells showed fewer beads were taken up into the cells, suggesting a lower phagocytic ability than non-activated M0 cells (*p* < 0.01). Treatment with FAM-inducers did not change significantly phagocytic activity within already M(LPS+/IFN-γ+) activated cells (*p* > 0.05).

### M(LPS+/IFN-γ+) Activated Macrophages Displayed Increased Lipid Content

**Figure 8:**
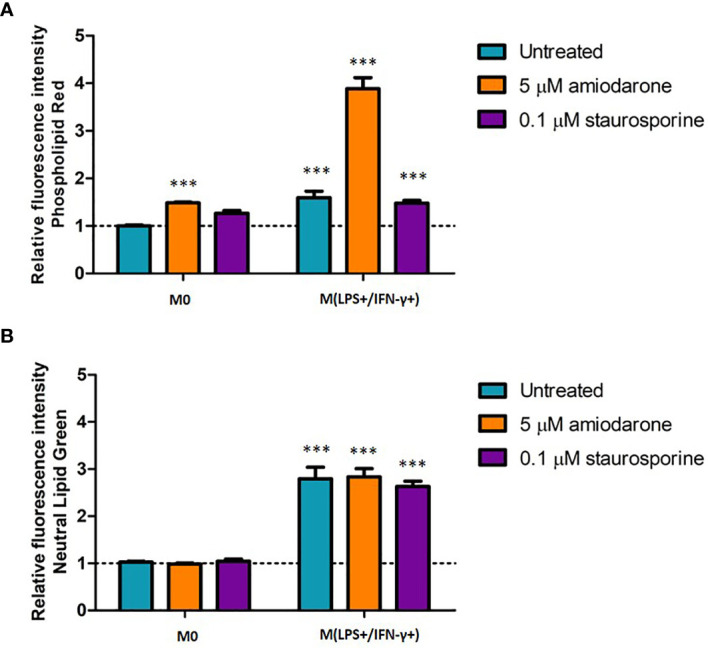
Phospholipid **(A)** and neutral lipid **(B)** content in M0-non-activated, LPS/IFN-γ and IL-4 activated NR8383 rat macrophages after 24 h exposure to 5 µM amiodarone (orange bars) and 0.1 µM staurosporine (purple bars). Data are normalized to cell count and presented as mean values ± SEM of three separate experiments. Statistical comparisons are made using a one-way ANOVA analysis with Bonferroni’s multiple comparison post-hoc test. Results are compared to non-polarized control (M0); *** indicates *p <* 0.001.

Lipid content was assessed in non-activated and activated macrophages after a drug challenge (**Figure 8**). Amiodarone-treated cells displayed 1.5-fold (*p* < 0.001) higher phospholipid accumulation in non-polarized cells, and 3.8 times (*p <*0.001) higher phospholipid content when cells where pre-activated to an M(LPS+/IFN-γ+) phenotype (**Figure 8A**). Neutral lipid content was significantly (*p* < 0.001) increased when cells were activated to M1 polarization prior to a drug treatment. There was no additional neutral lipid accumulation induced by amiodarone nor staurosporine when cells were M(LPS+/IFN-γ+) activated prior to a drug challenge (**Figure 8B**).

## Discussion

Macrophages are characterized by remarkable plasticity that can transform from one phenotype to another (2). These immune defense cells show various polarization states depending on micro-environmental stimuli and signals, and can be classified as classically activated (M1) or alternatively activated (M2) macrophages (2). M1-macrophages have anti-microbial and anti- tumor activities, mediate tissue damage and initiate inflammatory responses. They contribute to the host defense by generating reactive nitric oxide (NO) and releasing proinflammatory cytokines and chemokines such as IL-1β, IL-12, IL-23, CCL-2 and TNF-α. The generation of M1 macrophages is stimulated by such potent inducers as LPS and IFN-γ (1, 5, 14). M2-macrophages are implicated to play a vital role in immunosuppression, would healing or tumor progression (15). They can be activated by IL-4, IL-13, TGF-β and IL-10. Depending upon the specific stimuli, M2 macrophages can be further divided into M2a, M2b, M2c and M2d (16). However, there are no validated markers to distinguish between these subtypes. Phenotypically M1 macrophages present increased iNOS expression, while M2 macrophages are characterized by increased activity of Arg-1 enzyme, which in turn promotes collagen synthesis by making the amino acid proline available for fibroblasts (17). Therefore, NO production and arginase activity were chosen as M1 and M2 macrophage markers in this study, respectively, alongside specific cell surface receptor expression in order to confirm macrophage polarization status. Macrophage polarization has been widely investigated previously with an attempt to explain the alveolar macrophage activation status in various diseases e.g. COPD, lung cancer (13, 18–20). However, there are no studies to date investigating FAM responses in the context of macrophage activation. The appearance of foamy alveolar macrophages during rat *in vivo* inhalation therapy development studies is common and it is not certain is these changes observed in rats and other animal species indicate adversity to human lung health (6, 7). We have previously described FAM morphometry in different species and macrophage responses to amiodarone and/or staurosporine (8, 10). This study investigated the functionality of alveolar macrophages when polarized, and when induced to foamy phenotype providing new insights to understanding alveolar macrophage biology and their role in responding to xenobiotics.

First, we examined macrophage activation by quantification of NO cellular production and arginase-1 activity as markers for M1 or M2 activation, respectively. In this study, NR8383 cells were activated with a cytokine mix (LPS/IFN-γ) suggesting M1 polarization (**Figure 1A**). However, these results should be further confirmed by additional M1-markers evaluation. Therefore, in this study, LPS/IFN-γ-activated cells were referred as M(LPS+/IFN-γ+). While M2 activation was not achieved, with no increase in arginase-1 activity observed or increased expression of MRC-1 (**Figure 1B**). Whilst IL-4 at concentration 25 ng/mL has been shown to induce an M2 phenotype in various types of macrophages (e.g. monocyte-derived macrophages) (5), IL-4 was not sufficient for macrophage polarization of alveolar macrophages NR8383. To confirm these findings, MRC-1 receptor expression was quantified showing insignificant upregulation of this M2 polarization marker (**Figure 1C**). Furthermore, stimulation with both IL-4 and IL-13 also failed to polarize NR8383 to M2 status. Overall, in this study NR8383 rat alveolar macrophages stimulated with IL-4 or a mixture of IL-4 and IL-13 did not show a distinct M2 polarization.

Second, morphological features in the non-activated (model M0) and activated [model M(LPS+/IFN-γ+) or model M(IL-4+)] macrophages were characterized. M(LPS+/IFN-γ+) activated macrophages displayed distinct morphology (**Figure 2**). The cell area of macrophages activated with LPS/IFN-γ was increased, whilst the nuclear area was decreased when both parameters were compared with M0 cells. M(LPS+/IFN-γ+) cells showed an increased vacuolation pattern which was reflected in the increase of all vacuolation metrics (vacuoles number, average and total area of vacuoles, cellular area occupied by vacuoles). Distinct morphological features of M(LPS+/IFN-γ+) and M(IL-4+) macrophages have been recognized previously by Bertani et al. (21), where based only on qualitative fluorescence microscopy images, M(LPS+/IFN-γ+) macrophages were described to be of a spindle shape, while M(IL-4+) activated cells were more spread and very often multinucleated (21). However, no detailed morphological features have been measured. Our study for the first time provides a detailed quantification of M(LPS+/IFN-γ+) activated macrophage morphology (**Figures 2**, **3** and **Supplementary Figure 2**). Morphological analysis of cells is increasingly important as it aids examination of cell behavior. For example, recent developments of cell physiology and cell image analysis have begun to establish correlations between cell geometry and the activation of specific pathways in some cancers (22). Likewise, changes in alveolar macrophage physiology can be reflected in macrophage morphology, as demonstrated in this study for M(LPS+/IFN-γ+) activated cells.

Observed differences in M(LPS+/IFN-γ+) morphology were also reflected in cell function (**Figure 3**). Phagocytosis is a mechanism by which macrophages engulf and eliminate foreign particles. In this study, M(LPS+/IFN-γ+) macrophage activation affected their phagocytic capability. It has been reported previously that IFN-γ stimulation down-regulated phagocytosis (14). In line with that report, suppressed phagocytic activity was observed in M(LPS+/IFN-γ+) activated cells compared to M0. The accumulation of lipids (lipidosis) in macrophages is typically linked with macrophage dysfunction (23). However, the correlation between alveolar macrophage lipidosis and polarization has not previously been investigated. This study reports for the first time that, in addition to the characteristic increase in NO production, M(LPS+/IFN-γ+) cells had accumulated a pool of significantly more phospholipids and neutral lipids than non-activated control. This finding supports previously reported observations linking lipid-rich macrophages with the secretion of multiple pro-inflammatory mediators (23).

In contrast to the M(LPS+/IFN-γ+) distinct phenotypic changes, attempts to achieve M(IL-4)-activation did not affect cell morphology nor functionality, which confirms the failure of IL-4 to activate NR8383. This study did not show M(IL-4+) macrophages being multinucleated in response to stimulation with IL-4, as it has been described previously by Bertani et al. (21). This discrepancy in findings may be due to interspecies differences, highlighting variances in both polarization which further questions the relevance of animal models of inflammation. Furthermore, the source of macrophages may be an important consideration. Bertani et al. investigated macrophages derived from blood monocytes, while this study is focused specifically on alveolar macrophages. It has been demonstrated that populations of tissue-specific macrophages derived from yolk sac or fetal liver possess different functionalities to monocyte-derived macrophages from peripheral blood (13). Therefore, alveolar macrophage characterization and responses may be different from circulating macrophages (13).

Thirdly, we investigated macrophage activation in the presence of compounds that induce a FAM phenotype. Amiodarone and staurosporine were used as model drugs to induce FAM phenotype *in vitro* (10). Both compounds at the selected concentrations induced morphological changes but did not significantly affect (*p* < 0.05) cell viability (9) (**Supplementary Figure 1A**) and cell health assessed by mitochondrial activity and cell membrane permeability (**Supplementary Figure 1B**). Amiodarone is an established inducer of phospholipidosis and has been shown to induce a very distinct foamy macrophage phenotype typically characterized by a coarsely vacuolated appearance (24, 25). Staurosporine is pro-apoptotic agent reported previously to induce morphological foamy-like changes in a cell (26, 27).

Detailed individual macrophage responses characterized including cell viability, morphology and lipid content parameters have been described previously by Hoffman et al. (9, 10). The current study evaluated how these parameters are altered in the context of macrophage polarization and explored how pharmacological stimuli influence polarization status. Resident alveolar macrophages present in healthy lungs reside in the airways predominantly as a non-polarized population showing neither M1 nor M2 polarization (13). Therefore, the model M0 represents healthy alveolar macrophages, where NR8383 were only exposed to the foamy inducers, without pre-activation with cytokines. Patel et al. showed in the recent publication that amiodarone induced phospholipidosis in rat studies accompanied by increased inflammation (24). The current study confirms a similar effect of amiodarone *in vitro*. Increased phospholipid accumulation was observed, as well as increased NO production suggesting that macrophages are in proinflammatory state.

In contrast, staurosporine increased arginase activity while failing to induce accumulation of neutral lipids or phospholipids in M0 cells and appeared to direct a cell towards an anti-inflammatory and would healing phenotype. This finding is in agreement with established cell responses to staurosporine (26). In additional to being a potent proapoptotic agent, staurosporine also has antimicrobial, antifungal and immunosuppressive activity when dosed in sublethal concentrations (26). Furthermore, treatment with FAM-inducers, such as amiodarone and staurosporine, in sub-toxic doses did not modulate phagocytic activity, suggesting that although morphological changes in the cell occurred, these did not alter phagocytic processes. This important finding shows that macrophages preserve functionality even when displaying foamy morphology. The morphological observations reported in this study are easily defined and quantified using high content image analysis and have potential to provide an earlier and more sensitive indication of cell adaptation/adversity than biochemical and functional alternations. While adverse macrophage responses are typically linked with functional impairment (28, 29), morphological changes have the potential to determine whether a response is truly adverse or adaptive informing better go-no-go decision making in early pre-clinical safety assessment.

Finally, we evaluated FAM responses in a diseased *in vitro* model. Model M(LPS+/IFN-γ+) represented macrophages of inflamed lungs (e.g. in such diseases as asthma, COPD). M(LPS+/IFN-γ+) cells are the major effector macrophages in non-allergic asthma (30) and have been linked with the pathology of severe asthma and COPD (13, 17). NR8383 were activated with LPS and IFN-γ to M(LPS+/IFN-γ+) before drug exposure. We presented above that M(LPS+/IFN-γ+)-macrophages display a unique morphology. M(LPS+/IFN-γ+)-macrophages treated with amiodarone showed an elevated production of NO (one of M1-polarization marker), enhanced ability to accumulate phospholipids and reduced phagocytic capability. Staurosporine also increased NO production in M(LPS+/IFN-γ+) cells, as well as inducing increased production of lipids, not observed when M0 cells were treated with staurosporine. Overall, both FAM inducer compounds increased proinflammatory properties of macrophages in an already inflamed microenvironment.

It has been recognized that M1/M2 paradigm with its polarized extremes is oversimplification. However, these types of *in vitro* studies may provide a useful guide for studying macrophage biology *in vivo*. Likewise, macrophages placed in the culture may no longer resemble these which exist *in vivo*. Further studies should investigate how to assess macrophage phenotype *in vitro* more accurately.

In conclusion, we showed that M(LPS+/IFN-γ+) activated phenotype is reflected in altered cell morphology, which is manifested by an increased vacuolation pattern accompanied by increased phospholipid and neutral lipid accumulation. The high content image analysis technique was sufficiently sensitive to distinguish changes in polarized macrophages. Therefore, this technique could be implemented in safety assessment protocols in early *in vitro* preclinical studies facilitating decision making in drug discovery. These findings provide a new insight into foamy alveolar macrophage responses. Studying the detailed morphological changes within cells has the potential to determine whether a response is truly adverse or adaptive, transforming go-no-go decision-making, processes in early *in vitro* pre-clinical safety assessment.

## Data Availability Statement

The raw data supporting the conclusions of this article will be made available by the authors, without undue reservation.

## Author Contributions

EH, DM, and VH study conceptualization. EH designed the *in vitro* experiments. EH, RM, and PN performed the *in vitro* experiments and analyzed data. EH and VH, data visualization. EH, writing—original draft preparation. VH and DM, writing, review and editing. DM and VH, supervision. All authors contributed to the article and approved the submitted version.

## Funding

This work was supported by the Hertfordshire Science Partnership, an initiative part-financed by the Hertfordshire Local Enterprise Partnership’s “Growth Deal 2” under the Single Local Growth Fund Settlement.

## Conflict of Interest

The authors declare that the research was conducted in the absence of any commercial or financial relationships that could be construed as a potential conflict of interest.

## Publisher’s Note

All claims expressed in this article are solely those of the authors and do not necessarily represent those of their affiliated organizations, or those of the publisher, the editors and the reviewers. Any product that may be evaluated in this article, or claim that may be made by its manufacturer, is not guaranteed or endorsed by the publisher.

## Abbreviations

Arg-1, arginase 1; COPD, chronic obstructive pulmonary disease; FAM, foamy alveolar macrophage; FBS, fetal bovine serum; IFN-γ, interferon gamma; IL, interleukin; iNOS, inducible nitric oxide synthase; K-F12, Kaighn’s modified Ham’s F12 medium; LPS, lipopolysaccharide; M0, non-polarized/non-activated macrophages; M1, classically polarized macrophages; M2, alternatively polarized macrophage; M(LPS+/IFN-γ+), lipopolysaccharide and interferon gamma activated macrophages; M(IL-4+), interleukin-4 activated macrophages; MRC-1, mannose receptor c-type 1; NO, nitric oxide; NR8383, rat alveolar macrophage cell line; PBS, phosphate buffered saline; PFA, paraformaldehyde; TNF-α, tumor necrosis factor alpha; TGF-β, transforming growth factor beta.
